# Supplementary figures and images for: Antimicrobial Activity of Biogenic Metal Oxide Nanoparticles and Their Synergistic Effect on Clinical Pathogens
Source: Int J Mol Sci. 2023 Jun 10;24(12):9998. doi: 10.3390/ijms24129998 (PMC10298676; doi:10.3390/ijms24129998)

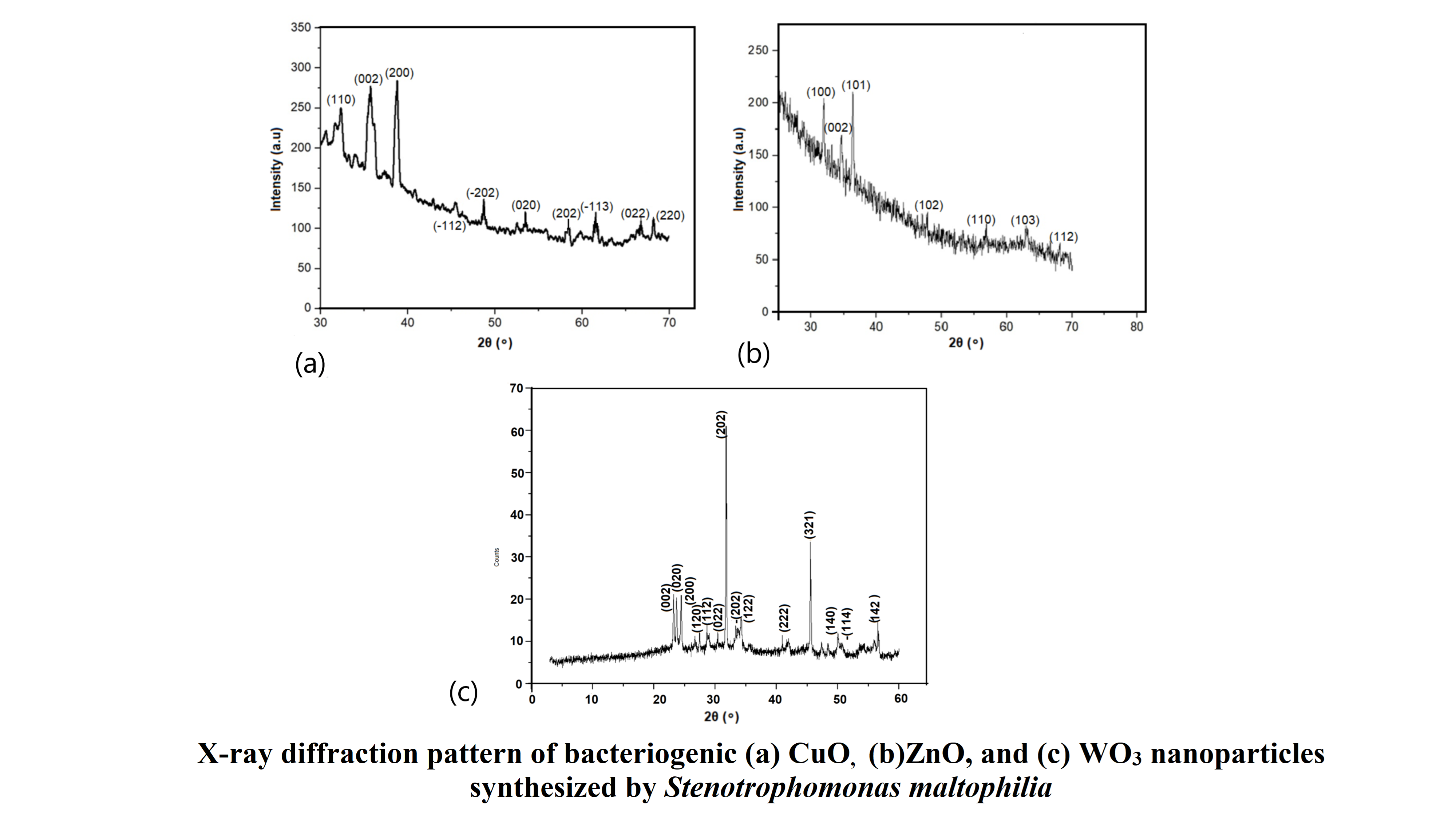

Supplement: Supplementary file 1 [file ijms-24-09998-s001.zip › Figure S1.tif]

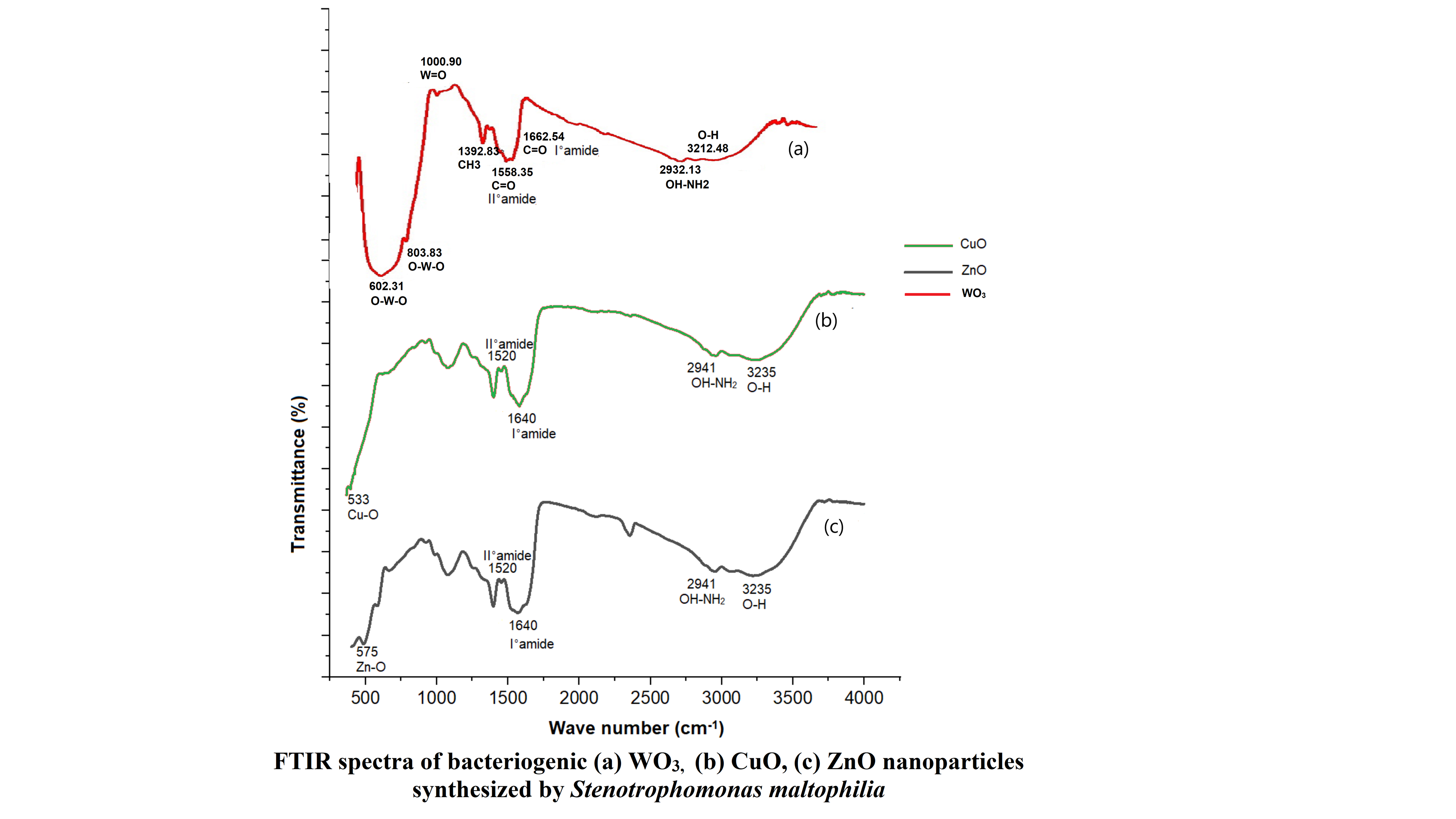

Supplement: Supplementary file 1 [file ijms-24-09998-s001.zip › Figure S2 .tif]

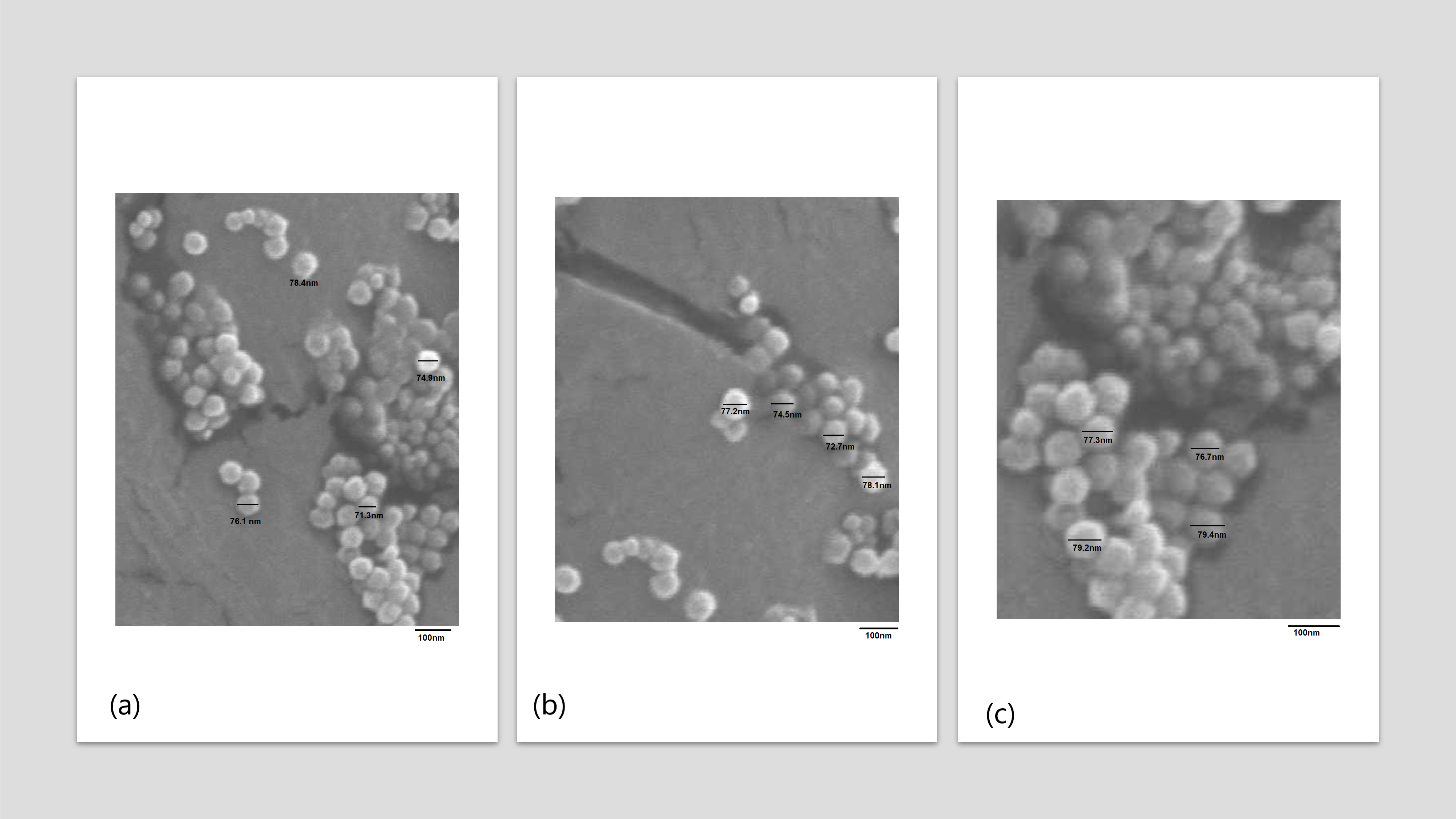

Supplement: Supplementary file 1 [file ijms-24-09998-s001.zip › Figure S3 SEM image.tif]
